# Investigation of CBX4 Polymorphisms and Their Association with Clinicopathological Features in Asian Patients with Oral Squamous Cell Carcinoma

**DOI:** 10.7150/jca.116232

**Published:** 2025-07-04

**Authors:** Li-Chung Hung, Yi-Chung Chien, Chiao-Wen Lin, Chun-Yi Chuang, Hsiu-San Hsu, Liang-Chih Liu, Shun-Fa Yang, Yung-Luen Yu

**Affiliations:** 1 Institute of Medicine, Chung Shan Medical University, Taichung 40201, Taiwan.; 2 Department of Radiation Oncology, Changhua Christian Hospital, Changhua 500, Taiwan.; 3 Institute of Translational Medicine and New Drug Development, China Medical University, Taichung 406040, Taiwan.; 4 Graduate Institute of Biomedical Sciences, China Medical University, Taichung 406040, Taiwan.; 5 Center for Molecular Medicine, China Medical University Hospital, Taichung 404327, Taiwan.; 6 Institute of Oral Sciences, Chung Shan Medical University, Taichung 40201, Taiwan.; 7 School of Medicine, Chung Shan Medical University, Taichung 40201, Taiwan.; 8 Department of Otolaryngology, Chung Shan Medical University Hospital, Taichung 40201, Taiwan.; 9 Department of Otolaryngology Head and Neck Surgery China Medical University Hospital, Taichung 404327, Taiwan.; 10 School of Medicine, College of Medicine, China Medical University, Taichung 40402, Taiwan.; 11 Department of Surgery, China Medical University Hospital, Taichung 404327, Taiwan.; 12 Department of Medical Research, Chung Shan Medical University Hospital, Taichung 40201, Taiwan.; 13 Department of Medical Laboratory Science and Biotechnology, Asia University, Taichung 413305, Taiwan.

**Keywords:** oral squamous cell carcinoma, *CBX4*, cancer progression, single-nucleotide polymorphisms

## Abstract

Head and neck squamous cell carcinomas (HNSCCs) are derived from the mucosal epithelium in the oral cavity, pharynx and larynx, and are predominantly linked to behavioral risk factors such as tobacco use and excessive alcohol consumption. In Taiwan, oral squamous cell carcinoma (OSCC) is one of the most prevalent subtypes of HNSCC and ranks as the fifth leading cause of cancer-related mortality in the country. The Chromobox (CBX) gene family encodes core subunits of the canonical Polycomb Repressive Complex 1 (cPRC1), a key epigenetic regulator mediating chromatin compaction and transcriptional silencing through histone modification. Dysregulation of CBX gene expression, whether at the transcriptional or post-transcriptional level, has been increasingly linked to tumorigenesis, metastasis, and cancer recurrence in a variety of malignancies. In particular, CBX4 has been implicated in the progression of gastric cancer, with growing evidence suggesting its broader role in cancer development. From the TCGA database, we found that levels of *CBX4* are lower in Asian OSCC patients compared with other races. This prompted us to further investigate the genetic landscape of *CBX4* in oral cancer. We conducted a case-control study involving 1184 male OSCC patients and 1188 healthy controls to evaluate whether genetic variants in* CBX4*, in conjunction with environmental exposures, contribute to OSCC susceptibility and progression in an Asian population. Four single-nucleotide polymorphisms (SNPs) of *CBX4* (rs1285251, rs2289728, rs3764374 and rs77447679) were selected for genotyping using real-time PCR. After adjusting for confounding factors such as age, smoking, alcohol intake, and betel-nut chewing, our analysis revealed that individuals harboring the CC or CT genotype at rs3764374 had a significantly increased risk of developing OSCC compared to those with the wild-type genotype. Notably, among male patients who had no history of betel-quid chewing, those carrying the CC/CT genotype at rs3764374 or the CA/AA genotype at rs77447679 exhibited a greater likelihood of presenting with stage II or III OSCC, indicating more advanced disease. These findings support the notion that specific *CBX4* polymorphisms may contribute to the pathogenesis and clinical progression of OSCC, particularly in populations exposed to distinct environmental risk factors. This study provides new insights into the gene-environment interplay in OSCC and suggests potential biomarkers for risk stratification and disease monitoring in Asian male populations.

## Introduction

Head and neck squamous cell carcinomas (HNSCCs) represent a heterogeneous group of epithelial malignancies that arise from the mucosal linings of the oral cavity, nasopharynx, oropharynx, hypopharynx, and larynx, with squamous cell histology being the predominant pathological subtype. Globally, HNSCC ranks as the sixth most prevalent cancer, with oral squamous cell carcinoma (OSCC) constituting a major clinical subtype 1-4. Although advances in multimodal therapeutic strategies, including surgery, radiotherapy, and chemotherapy, have improved disease management, the overall five-year survival rate remains unsatisfactory, ranging between 30% and 50%, largely due to frequent locoregional recurrence and distant metastatic dissemination 5, 6. The etiology of OSCC is multifactorial, involving cumulative genetic and epigenetic alterations triggered by prolonged exposure to environmental carcinogens. Key contributing risk factors include persistent mucosal inflammation, tobacco and alcohol use, areca (betel) nut chewing, and oncogenic viral infections. The Chromobox (CBX) gene family encodes core subunits of the canonical Polycomb Repressive Complex 1 (cPRC1), a key epigenetic regulator mediating chromatin compaction and transcriptional silencing through histone modification. Altered transcriptional or post-transcriptional regulation of CBX members has been increasingly associated with oncogenic processes across diverse malignancies.

Reports suggest that CBX3 has been identified as a key player in lung cancer progression by interacting with pathways such as phosphoinositide 3-kinase (PI3K)/ protein kinase B (AKT), rat sarcoma virus (Ras)/ kirsten rat sarcoma virus (KRAS), Wnt/β-catenin, mitogen-activated protein kinase (MAPK), Notch, and p53, leading to increased proliferation and therapy resistance 7. Similarly, CBX2 overexpression has been associated with poor survival outcomes in cancer patients, potentially by maintaining cancer stem cells in an undifferentiated state and repressing tumor suppressor genes 8. In ovarian cancer, components of PRC1, including CBX proteins, contribute to disease development, recurrence, and chemoresistance by silencing tumor suppressor genes and regulating stem cell compartments 8. The diverse roles of CBX proteins in cancer underscore their potential as therapeutic targets. Understanding the specific functions and mechanisms of each CBX member is crucial for developing targeted cancer therapies 9. CBX4, a pivotal component of the PRC1, plays multifaceted roles in cancer development and progression. Its functions vary across cancer types, exhibiting both oncogenic and tumor-suppressive activities. ​In colorectal carcinoma, CBX4 suppresses metastasis by recruiting histone deacetylase 3 (HDAC3) to the runt-related transcription factor 2 (Runx2) promoter, leading to transcriptional repression of Runx2, a key factor in cancer metastasis. This mechanism underscores the tumor-suppressive role of CBX4 in this context 10. Conversely, in breast cancer, CBX4 exhibits oncogenic properties by downregulating miR-137, resulting in the activation of the Notch1 signaling pathway, which promotes tumor progression 11. Furthermore, CBX4 has been implicated in colon cancer development through its potential influence on circadian rhythm and immune infiltration, suggesting a complex role in tumorigenesis 12. ​Although these studies collectively demonstrate that the function of CBX4 in cancer is context-dependent, acting as either an oncogene or tumor suppressor depending on the cancer type and molecular environment. Its mechanistic contribution to OSCC development and progression remains insufficiently characterized.

Recent studies have identified various genetic susceptibility factors that may contribute to the development of head and neck squamous cell carcinoma (HNSCC). Among these, single-nucleotide polymorphisms (SNPs), the most common type of genetic variation, have shown significant potential as predictive biomarkers for assessing individual cancer risk 13-15. By modulating transcriptional activity or protein structure, SNPs can impact cellular pathways critical to tumor initiation and development. Recent evidence has identified a significant association between the *CBX4* rs77447679 polymorphism and the risk of gastric cancer. CBX4 encodes a component of the PRC1 and also functions as a SUMO E3 ligase, implicating it in chromatin modification and post-translational regulation, both of which are critical in cancer development. The observed correlation between rs77447679 and gastric cancer risk underscores the potential biological relevance of CBX4-mediated sumoylation in gastrointestinal malignancies​ 16. However, to date, no studies have specifically addressed the role of CBX4 SNPs in OSCC. Therefore, our present study aims to explore this previously uncharacterized association and provide novel insights into the genetic determinants of OSCC pathogenesis.

## Materials and Methods

### Study participants and specimen collection

This study enrolled a total of 1,184 male patients diagnosed with oral squamous cell carcinoma (OSCC) from Chung Shan Medical University Hospital in Taichung, Taiwan. All participants provided written informed consent after receiving a comprehensive explanation of the study protocol. Clinical staging for OSCC was determined at diagnosis according to the tumor-node-metastasis (TNM) classification system established by the American Joint Committee on Cancer (AJCC, 2002). A comparison group of 1,188 cancer-free individuals, aged 20 to 70, was recruited from the Taiwan Biobank (https://www.twbiobank.org.tw/new_web_en/index.php). Detailed information regarding age, sex, and lifestyle behaviors (including betel nut chewing, tobacco use, and alcohol intake) was collected through structured interviews. Participants who reported drinking more than two alcoholic beverages daily were categorized as alcohol users, while those who had smoked at least one cigarette per day within the past three months were identified as habitual smokers. Ethical approval for this research was granted by the Institutional Review Board of Chung Shan Medical University Hospital.

### Comprehensive analysis of *CBX4* from The Cancer Genome Atlas (TCGA)

The UALCAN platform (http://ualcan.path.uab.edu/index.html) offers an intuitive and interactive interface for exploring cancer transcriptomics and clinical datasets. It leverages level 3 RNA sequencing and associated clinical information from The Cancer Genome Atlas (TCGA), covering 31 distinct tumor types 17. In this study, UALCAN was utilized to assess CBX4 expression differences between tumor and normal tissues, as well as to evaluate its prognostic relevance based on overall survival in patients with HNSCC. Bioinformatics analyses were conducted using UALCAN, platforms known for its validated and reproducible methodologies in cancer research 17.

### Identifying gene set enrichments of CBX4 in TCGA database

Large-scale initiatives such as TCGA have generated extensive genomic profiles linked to various tumor types. While several online tools have been developed to facilitate data access, many still require a foundational understanding of bioinformatics to perform more advanced analyses. To investigate the gene set enrichment associated with CBX4 expression within TCGA datasets, we utilized the Gene ENrichment Identifier (GENI; https://www.shaullab.com/geni), a web-based resource that efficiently computes transcriptome-wide correlations for a target gene and ranks them according to curated biological gene sets 18.

### Selection of *cbx4* polymorphisms

In this investigation, four SNPs within the *CBX4* (NM_003655.3) were selected based on data from the International HapMap Project: rs1285251, rs2289728, rs3764374, and rs77447679. These SNPs were selected to investigate their potential roles in modulating CBX4 expression and function, which may contribute to disease susceptibility and pathogenesis.

### *CBX4* genotyping

Genotyping of the *CBX4* variants rs1285251, rs2289728, rs3764374, and rs77447679 was performed using the TaqMan allelic discrimination method on the ABI StepOne Real-Time PCR platform (Applied Biosystems). The reactions were analyzed with SDS software version 3.0, following the manufacturer's protocol to ensure accurate detection of each SNP 13.

### Statistical analysis

Differences in age and baseline demographic variables between OSCC cases and control participants were assessed using the nonparametric Mann-Whitney U test. Logistic regression analysis was conducted to calculate odds ratios (ORs) and corresponding 95% confidence intervals (CIs) to determine the strength of associations. A threshold of *p* < 0.05 was applied to define statistical significance. All analyses were performed using SAS software.

## Results

**Figure 1:**
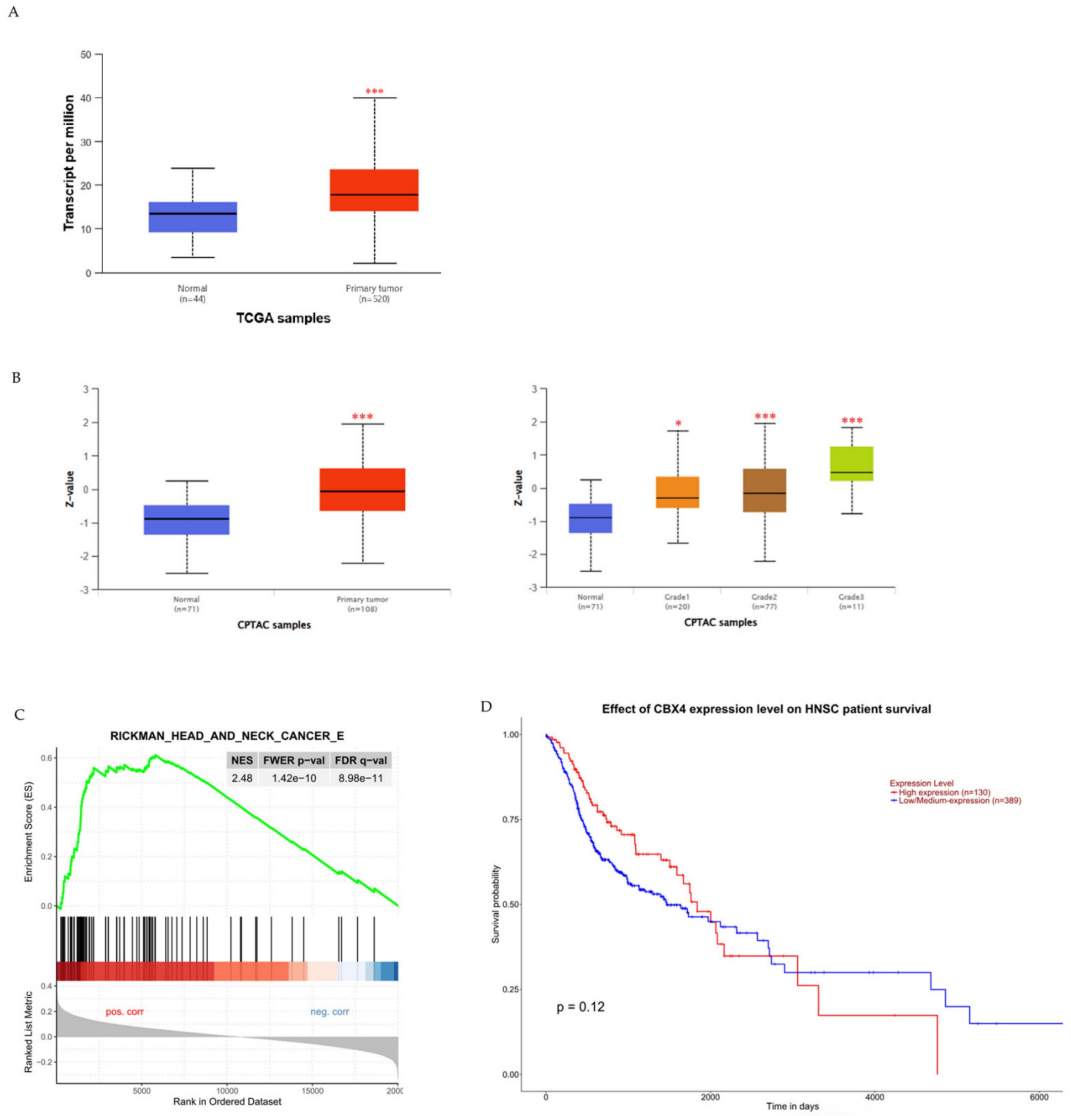
** Association of *CBX4* expression with HNSCC progression and patient survival outcomes. (A)** Comparative analysis of *CBX4* transcript levels in normal tissues versus HNSCC patient samples, demonstrating a significant elevation in tumor tissues. **(B)** Quantification of CBX4 protein expression across different histological grades of HNSCC, revealing progressively increased levels in grade 2 and grade 3 tumors compared to normal controls. **(C)** Gene Set Enrichment Analysis (GSEA) plot illustrating a strong enrichment of head and neck cancer-related gene signatures in samples with high CBX4 expression, suggesting a potential role in promoting malignant transformation. **(D)** Kaplan-Meier survival curves comparing overall survival among HNSCC patients stratified by *CBX4* expression levels, based on UALCAN database analysis. * p < 0.05, ** p < 0.01, *** p < 0.001.

To examine the potential clinical significance of *CBX4* expression in HNSCC, we utilized the UALCAN platform to compare *CBX4* mRNA and protein levels between healthy controls and HNSCC patients, and to investigate any associations with patient survival. Initially, our analysis revealed a significant elevation in *CBX4* transcript levels among HNSCC patients compared to control individuals (p < 0.001), indicating a possible oncogenic role for CBX4 in the progression of HNSCC (Figure 1A). Additionally, to further substantiate these findings, we assessed CBX4 protein expression utilizing data from the Clinical Proteomic Tumor Analysis Consortium (CPTAC). Protein abundance, expressed as Z-scores normalized against median values from normal samples, demonstrated significantly higher CBX4 protein levels within tumor tissues, particularly in moderately (grade 2) to poorly differentiated (grade 3) tumors (Figure 1B). Moreover, subsequent gene enrichment analysis indicated significant enrichment of head and neck cancer-related pathways in the group characterized by high CBX4 expression (Normalized Enrichment Score, NES = 2.48), suggesting the involvement of CBX4 in promoting aggressive tumor behavior (Figure 1C). Interestingly, despite the clear association between elevated *CBX4* expression and aggressive HNSCC phenotypes, our survival analysis indicated no statistically significant impact of *CBX4* expression levels on overall patient survival (p = 0.12, Figure 1D). Collectively, these results imply complex regulatory mechanisms of *CBX4* in HNSCC progression that remain to be fully elucidated. Further research should be directed toward uncovering the molecular pathways through which CBX4 contributes to tumor aggressiveness, independently of survival outcomes.

To explore potential risk factors contributing to the onset of OSCC in a clinical context, we conducted a case-control study involving 1184 male patients diagnosed with OSCC and 1188 matched healthy male controls. Comparative analysis of demographic and lifestyle factors between these two groups revealed several significant differences. Specifically, OSCC patients were notably older than healthy controls (p < 0.001). Moreover, lifestyle habits traditionally linked to OSCC, such as betel-nut chewing, tobacco smoking, and alcohol intake, were significantly more prevalent among OSCC patients compared to controls (all p-values < 0.001, Table 1). These findings reinforce the critical roles of age and lifestyle behaviors as significant contributors to OSCC development, highlighting the necessity for targeted preventive interventions and health education to mitigate risk among susceptible populations. Future investigations should further elucidate interactions between genetic susceptibility and environmental exposures to enhance risk prediction and inform personalized treatment strategies.

To mitigate potential confounding effects arising from various environmental exposures, we performed multivariate logistic regression analyses adjusting for critical risk factors, including age, betel-quid chewing, tobacco smoking, and alcohol consumption. Adjusted odds ratios (AORs) along with their 95% confidence intervals (CIs) were calculated to accurately reflect the associations between CBX4 polymorphisms and OSCC. The detailed genotype distributions and their relationships to OSCC susceptibility are presented comprehensively in Table 2. Specifically, the most prevalent allelic genotypes identified in CBX4 polymorphisms rs1285251, rs2289728, rs3764374, and rs77447679 were homozygous C/C, homozygous G/G, homozygous C/C, and homozygous C/C, respectively, observed consistently among both OSCC cases and control participants. Nevertheless, statistical analysis indicated that carriers of these polymorphisms (rs1285251, rs2289728, rs3764374, and rs77447679) did not demonstrate a statistically significant altered risk of developing OSCC compared to individuals possessing the wild-type genotypes.

To further investigate potential clinical implications of CBX4 polymorphisms in OSCC, we analyzed associations between various CBX4 SNP genotypes and clinical characteristics of the patients. Our analysis highlighted one particular SNP, rs3764374, that appears to influence OSCC disease progression. Specifically, patients harboring the variant genotypes (C/T or T/T) of the rs3764374 polymorphism exhibited a significantly elevated risk of developing OSCC compared to those with the wild-type C/C genotype (AOR = 1.434; 95% CI: 1.02-2.012; p = 0.037, Table 3). This finding suggests that the rs3764374 variant of CBX4 could serve as a predictive genetic marker for OSCC susceptibility and might contribute to identifying individuals at greater risk of aggressive disease. Future research exploring the functional implications and underlying molecular mechanisms of this SNP would be valuable for enhancing early risk assessment and developing personalized therapeutic strategies for OSCC patients.

Betel nut chewing has consistently been identified as a crucial environmental factor influencing the onset and advancement of OSCC. To more precisely evaluate its impact in our study, we categorized OSCC patients into two distinct groups based on their history of betel nut chewing. Subsequently, we investigated the distribution of *CBX4* genotypes and explored potential correlations with clinicopathological features within these groups. Our results notably revealed that *CBX4* SNPs, specifically rs3764374 and rs77447679, exhibited significant associations with increased rates of OSCC progression exclusively among individuals who had never engaged in betel nut chewing (Tables 4 and 5). These findings suggest a complex interplay between genetic susceptibility and environmental influences, highlighting that genetic predisposition through *CBX4* variants may independently drive disease progression in the absence of traditional risk factors. Further studies are necessary to delineate the biological mechanisms underlying these genetic interactions, providing opportunities for tailored prevention strategies and targeted therapies in genetically susceptible populations.

## Discussion

Single-nucleotide polymorphisms (SNPs) represent common genetic variations occurring at a single nucleotide position in the human genome. Unlike rare mutations, SNPs are defined by their relatively high frequency within the population and are often shaped by environmental influences. These genetic variants contribute to phenotypic diversity and have been linked to an individual's predisposition to various diseases, including malignancies 19, 20. Alterations in gene function resulting from SNPs or other genetic changes can modify disease susceptibility and are associated with an elevated risk of cancer and distinct pathological features 21, 22.

CBX proteins, particularly CBX4, play pivotal roles in cancer biology through their involvement in epigenetic regulation. CBX4, a component of the PRC1, modulates gene expression by recognizing histone modifications and recruiting other epigenetic modifiers. In lung adenocarcinoma, CBX4 exhibits a dual function. It promotes tumor proliferation by upregulating phosphoglycerate dehydrogenase (PHGDH) expression via interaction with the histone acetyltransferase general control non-depressible 5 (GCN5), enhancing serine biosynthesis; concurrently, it suppresses metastasis by repressing zinc finger E-box binding homeobox 2 (ZEB2) transcription through PRC1-mediated H2AK119 ubiquitination 23. ​Genetic variations in CBX4, such as single nucleotide polymorphisms (SNPs), have been associated with cancer susceptibility and prognosis. For instance, the CBX4 rs2289728 (G>A) SNP has been linked to a decreased risk of hepatocellular carcinoma (HCC), potentially by reducing CBX4 expression and alleviating repression of tumor suppressor genes like cyclin-dependent kinase inhibitor 2A (CDKN2A). Conversely, the rs77447679 (C>A) SNP in CBX4 has been associated with increased gastric cancer risk and poorer survival in HCC patients, correlating with higher CBX4 expression and more aggressive tumor characteristics 16, 23, 24.​ CBX4 polymorphisms may modulate the regulatory functions of genes, influencing immune responses to environmental carcinogens. The role of CBX4 in suppressing programmed death-1 (PD-1) expression in T cells suggests a mechanism by which genetic and environmental factors could synergistically increase OSCC risk 25. Taken together, current evidence supports a critical role for CBX4 in cancer development and progression through both epigenetic regulation and genetic polymorphism. Despite ongoing research, the relationship between genetic variants of CBX4 and OSCC susceptibility remains poorly understood. However, CBX4 may contribute to OSCC progression by regulating genes involved in cell cycle and stemness, such as CDC20, as observed in gastric cancer studies 26. Further research is needed to elucidate these mechanisms in OSCC. Our investigation aims to elucidate how specific SNPs within CBX4 influence the risk and clinical manifestations associated with OSCC.

In Taiwan, head and neck cancer rates remain elevated primarily because of prevalent risk factors such as dietary patterns and the chewing of betel nuts. Our study revealed increased CBX4 expression at both the transcript and protein levels, indicating its potential involvement in promoting HNSCC progression (Figure 1). Although CBX4 expression is associated with aggressive tumor characteristics, its impact on overall survival may be influenced by treatment efficacy and other molecular factors, as observed in studies on clear cell renal cell carcinoma 17. The four CBX4 SNPs were selected based on their minor allele frequency (>0.05) in the Chinese population and potential functional significance, as identified through the NCBI dbSNP database and subsequent analyses 16. Interestingly, we discovered associations between specific CBX4 SNPs, rs3764374 (CT+TT genotype) and rs77447679, and more aggressive oral cancer progression, particularly among individuals without betel nut chewing habits (Tables 4 and 5). The absence of significant associations for rs1285251 and rs2289728 may be attributed to their limited impact on CBX4 function or expression, as opposed to other variants like rs77447679 which have demonstrated functional significance in cancer susceptibility 23. Our findings suggest that CBX4 polymorphisms, such as rs77447679, may contribute to OSCC progression particularly in individuals without betel quid exposure, highlighting a potential gene-environment interaction where genetic factors play a more pronounced role in the absence of environmental carcinogens 16. Nonetheless, further investigations are necessary to clarify the precise molecular pathways through which CBX4 SNPs influence OSCC development.

## Conclusion

In summary, our findings highlight the potential clinical relevance of *CBX4* genetic variants as predictive markers for susceptibility to OSCC. Our results offer novel insights into the relationship between CBX4 polymorphisms and the clinicpathological characteristics of OSCC in the Taiwanese population. These findings underscore the importance of genetic factors in OSCC pathogenesis and progression. Moreover, the identified SNPs in *CBX4* could contribute to improved screening and personalized therapeutic approaches for patients with OSCC. However, the homogeneity of the Taiwanese cohort reduces the likelihood of population stratification, we recognize that unaccounted stratifications may exist and could affect the findings. Future research focusing on the underlying molecular mechanisms and functional implications of these polymorphisms is warranted to validate their clinical utility and further clarify their role in cancer biology.

## Figures and Tables

**Table 1:** The distributions of demographical characteristics in 1188 controls and 1184 male patients with oral cancer.

Variable	Controls (N=1188)	Patients (N=1184)	*p* value
Age (yrs)			*p* = 0.028*
< 55	561 (47.2%)	506 (42.7%)	
≥ 55	627 (52.8%)	678 (57.3%)	
Betel quid chewing			*p* < 0.001*
No	990 (83.3%)	387 (32.7%)	
Yes	198 (16.7%)	797 (67.3%)	
Cigarette smoking			*p* < 0.001*
No	556 (46.8%)	249 (21.0%)	
Yes	632 (53.2%)	935 (79.0%)	
Alcohol drinking			*p* < 0.001*
No	952 (80.1%)	734 (62.0%)	
Yes	236 (19.9%)	450 (38.0%)	
Stage			
I+II		514 (43.4%)	
III+IV		670 (56.6%)	
Tumor T status			
T1+T2		580 (49.0%)	
T3+T4		604 (51.0%)	
Lymph node status			
N0		788 (66.6%)	
N1+N2+N3		396 (33.4%)	
Metastasis			
M0		1177 (99.4%)	
M1		7 (0.6%)	
Cell differentiation			
Well differentiated		186 (15.7%)	
Moderately or poorly differentiated		998 (84.3%)	

Mann-Whitney U test or Chi-square test was used between healthy controls and patients with oral cancer. * p value < 0.05 as statistically significant.

**Table 2:** Genotyping and allele frequency of *CBX4* single nucleotide polymorphism (SNP) in oral cancer and normal controls.

Variable	Controls (N=1188) n (%)	Patients (N=1184) n (%)	AOR (95% CI)^a^
**rs1285251**			
CC	525 (44.2%)	532 (44.9%)	1.000 (reference)
CT	532 (44.8%)	510 (43.1%)	0.990 (0.811-1.210)
TT	131 (11.0%)	142 (12.0%)	1.106 (0.811-1.510)
CT+TT	663 (55.8%)	652 (55.1%)	1.007 (0.916-1.107)
**rs2289728**			
GG	370 (31.1%)	394 (33.3%)	1.000 (reference)
GA	594 (50.3%)	556 (47.0%)	0.812 (0.655-1.005)
AA	221 (18.6%)	234 (19.7%)	0.956 (0.729-1.253)
GA+AA	818 (68.9%)	790 (66.7%)	0.922 (0.834-1.020)
**rs3764374**			
CC	742 (62.5%)	744 (62.8%)	1.000 (reference)
CT	396 (33.3%)	395 (33.4%)	1.044 (0.853-1.278)
TT	50 (4.2%)	45 (3.8%)	0.927 (0.568-1.511)
CT+TT	446 (37.5%)	440 (37.2%)	1.015 (0.921-1.119)
**rs77447679**			
CC	747 (62.9%)	746 (63.0%)	1.000 (reference)
CA	393 (33.1%)	398 (33.6%)	1.112 (0.909-1.361)
AA	48 (4.0%)	40 (3.4%)	0.770 (0.460-1.288)
CA+AA	441 (37.1%)	438 (37.0%)	1.036 (0.940-1.142)

The ORs with analyzed by their 95% CIs were estimated by logistic regression models. ^a^ Adjusted for the effects of age, betel quid chewing, cigarette smoking and alcohol drinking.

**Table 3:** Adjusted odds ratio (AOR) and 95% confidence intervals (CI) of clinical statuses associated with genotypic frequencies of *CBX4* rs3764374 in male oral cancer patients.

Variable	CC (N=744)	CT+TT (N=440)	AOR (95% CI)	*p* value
**Clinical stage**				
Stage I+II	319 (42.9%)	195 (44.3%)	1.000 (reference)	0.665
Stage III+IV	425 (57.1%)	245 (55.7%)	0.947 (0.746-1.202)	
**Tumor size**				
≤ T2	364 (48.9%)	216 (49.1%)	1.000 (reference)	0.985
> T2	380 (51.1%)	224 (50.9%)	1.002 (0.791-1.270)	
**Lymph node metastasis**				
No	486 (65.3%)	302 (68.6%)	1.000 (reference)	0.296
Yes	258 (34.7%)	138 (31.4%)	0.874 (0.678-1.125)	
**Cell differentiated grade**				
Grade I	129 (17.3%)	57 (10.0%)	1.000 (reference)	0.037^*^
Grade II or III	615 (82.7%)	383 (87.0%)	1.434 (1.021-2.012)	

Cell differentiated grade: grade I: well differentiated; grade II: moderately differentiated; grade III: poorly differentiated. The adjusted odds ratio (AOR) with their 95% confidence intervals were estimated by multiple logistic regression models after controlling for age, betel quid chewing, cigarette smoking, and alcohol drinking. * *p* value < 0.05 as statistically significant.

**Table 4:** Adjusted odds ratio (AOR) and 95% confidence intervals (CI) of clinical statuses associated with genotypic frequencies of *CBX4* rs3764374 in male oral cancer patients among with betel quid chewers or without betel quid chewers.

	Non-Betel Quid Chewers (N=387)				Betel Quid Chewers (N=797)			
Variable	CC (N=240)	CT+TT (N=147)	AOR (95% CI)	*p* value	CC (N=504)	CT+TT (N=293)	AOR (95% CI)	*p* value
**Clinical Stage**								
Stage I+II	99 (41.2%)	60 (40.8%)	1.000 (reference)	0.945	220 (43.7%)	135 (46.1%)	1.000 (reference)	0.519
Stage III+IV	141 (58.8%)	87 (59.2%)	1.015 (0.666-1.546)		284 (56.3%)	158 (53.9%)	0.909 (0.680-1.215)	
**Tumor size**								
≤ T2	115 (47.9%)	64 (43.5%)	1.000 (reference)	0.349	249 (49.4%)	152 (51.9%)	1.000 (reference)	0.494
> T2	125 (52.1%)	83 (56.5%)	1.220 (0.805-1.850)		255 (50.6%)	141 (48.1%)	0.904 (0.677-1.208)	
**Lymph node metastasis**								
No	158 (65.8%)	96 (65.3%)	1.000 (reference)	0.909	328 (65.1%)	206 (70.3%)	1.000 (reference)	0.169
Yes	82 (34.2%)	51 (34.7%)	1.026 (0.664-1.586)		176 (34.9%)	87 (29.7%)	0.803 (0.588-1.097)	
**Cell differentiated grade**								
Grade I	35 (14.6%)	11 (7.5%)	1.000 (reference)	0.036*	94 (18.7%)	46 (15.7%)	1.000 (reference)	0.252
Grade II or III	205 (85.4%)	136 (92.5%)	2.152 (1.051-4.406)		410 (81.3%)	247 (84.3%)	1.255 (0.851-1.850)	

Cell differentiated grade: grade I: well differentiated; grade II: moderately differentiated; grade III: poorly differentiated. The adjusted odds ratio (AOR) with their 95% confidence intervals were estimated by multiple logistic regression models after controlling for age, cigarette smoking, and alcohol drinking. * *p* value < 0.05 as statistically significant.

**Table 5:** Adjusted odds ratio (AOR) and 95% confidence intervals (CI) of clinical statuses associated with genotypic frequencies of *CBX4* rs77447679 in male oral cancer patients among with betel quid chewers or without betel quid chewers.

	Non-Betel Quid Chewers (N=387)				Betel Quid Chewers (N=797)			
Variable	CC (N=233)	CA+AA (N=154)	AOR (95% CI)	*p* value	CC (N=513)	CA+AA (N=284)	AOR (95% CI)	*p* value
**Clinical Stage**								
Stage I+II	100 (42.9%)	59 (38.3%)	1.000 (reference)	0.394	224 (43.7%)	131 (46.1%)	1.000 (reference)	0.506
Stage III+IV	133 (57.1%)	95 (61.7%)	1.201 (0.788-1.830)		289 (56.3%)	153 (53.9%)	0.905 (0.676-1.213)	
**Tumor size**								
≤ T2	114 (48.9%)	65 (42.2%)	1.000 (reference)	0.145	259 (50.5%)	142 (50.0%)	1.000 (reference)	0.939
> T2	119 (51.1%)	89 (57.8%)	1.363 (0.899-2.067)		254 (49.5%)	142 (50.0%)	1.011 (0.755-1.354)	
**Lymph node metastasis**								
No	157 (67.4%)	97 (63.0%)	1.000 (reference)	0.390	334 (65.1%)	200 (70.4%)	1.000 (reference)	0.169
Yes	76 (32.6%)	57 (37.0%)	1.209 (0.784-1.862)		179 (34.9%)	84 (29.6%)	0.802 (0.586-1.099)	
**Cell differentiated grade**								
Grade I	34 (14.6%)	12 (7.8%)	1.000 (reference)	0.039*	94 (18.3%)	46 (16.2%)	1.000 (reference)	0.402
Grade II or III	199 (85.4%)	142 (92.2%)	2.094 (1.040-4.219)		419 (81.7%)	238 (83.8%)	1.181 (0.800-1.743)	

Cell differentiated grade: grade I: well differentiated; grade II: moderately differentiated; grade III: poorly differentiated. The adjusted odds ratio (AOR) with their 95% confidence intervals were estimated by multiple logistic regression models after controlling for age, cigarette smoking, and alcohol drinking. * *p* value < 0.05 as statistically significant.
